# Changes of choroidal structure after treatment for primary intraocular lymphoma: retrospective, observational case series

**DOI:** 10.1186/s12886-015-0127-7

**Published:** 2015-10-19

**Authors:** Mariko Egawa, Yoshinori Mitamura, Hiroki Sano, Kei Akaiwa, Masanori Niki, Kentaro Semba, Shozo Sonoda, Taiji Sakamoto

**Affiliations:** Department of Ophthalmology, Institute of Biomedical Sciences, Tokushima University Graduate School, 3-18-15 Kuramoto, Tokushima, 770-8503 Japan; Department of Ophthalmology, Kagoshima University Graduate School of Medical and Dental Sciences, Kagoshima, Japan

**Keywords:** Binarization, Choroidal structure, Enhanced depth imaging optical coherence tomography, Intravitreal methotrexate injection, Primary intraocular lymphoma

## Abstract

**Background:**

We report changes of choroidal structure determined by binarization of enhanced depth imaging optical coherence tomographic (EDI-OCT) images after treatment for primary intraocular lymphoma (PIOL).

**Methods:**

Five eyes of four patients with PIOL were examined by EDI-OCT before and 6 months after intravitreal methotrexate injections. In addition, 15 eyes of 15 normal individuals controlled by age and refractive error were examined by EDI-OCT. Binarization of the EDI-OCT images was performed using publicly accessible software (ImageJ). The examined area of the subfoveal choroid was 1,500 μm wide, and the dark areas that represented the luminal areas were traced by the Niblack method. Wilcoxon signed rank test was used to determine the significance of changes in the subfoveal choroidal thickness, interstitial area, and luminal area. Mann–Whitney *U* test was used to compare the parameters in the eyes with pretreatment PIOL and normal control eyes.

**Results:**

The subfoveal choroidal thickness was significantly decreased after treatment (*P* = 0.0431). In the binarized images, the interstitial area was significantly decreased after treatment (*P* = 0.0431), while the luminal area was not significantly changed (*P* = 0.8927). After delayed onset of PIOL, increased interstitial area, thickened choroid and unchanged luminal area were observed in one eye. The interstitial area and choroidal thickness were significantly increased in the eyes with pretreatment PIOL compared with the normal control eyes (*P* = 0.0207, *P* = 0.0495, respectively), while the luminal area was not significantly different (*P* = 0.2752).

**Conclusions:**

After treatment for PIOL, the EDI-OCT images showed a thinner choroid, and binarization of the EDI-OCT images showed significantly decreased interstitial areas compared with the luminal areas. The binarized EDI-OCT images can provide useful information on choroidal structure in eyes with PIOL, and combining these images with intraocular interleukin levels or fundus autofluorescence images should provide valuable information for determining the PIOL activity.

## Background

Intraocular lymphoma has two distinct forms: (1) arising in the central nervous system (CNS) and/or the retina and (2) arising outside the CNS with intraocular metastasis [1, 2]. The latter is a non-primary CNS lymphoma (non-PCNSL), and originates from systemic lymphomas that metastasize via blood circulation to the uvea. The former is called primary CNS lymphoma (PCNSL), and has a much higher prevalence than non-PCNSL. Primary intraocular lymphoma (PIOL) is a type of PCNSL. They are generally classified as aggressive, diffuse, large B-cell lymphomas. It has been reported that 15 % to 25 % of PCNSL cases involved the eye, but PIOLs could be present without CNS involvement [3, 4]. Patients with a PIOL often have iritis, vitreous opacities, and retinal infiltrations, and are often misdiagnosed as having uveitis. The prognosis of PIOL remains poor with a high tendency to progress to PCNSL. Kimura et al. [5] reported that the 5-year survival rate of PIOL was 61.1 %. Therefore, it is important to diagnose PIOL promptly and to accurately evaluate the effects of treatment.

Optical coherence tomographic (OCT) findings have been recently reported to be helpful in determining the retinal abnormalities in eyes with PIOL [6–10]. We have reported retinal abnormalities on the OCT images in three cases of PIOL before treatment [11]. We have also reported that subfoveal choroidal thickness on the enhanced depth imaging OCT (EDI-OCT) images, gradually decreased in one case during the treatment for PIOL [12]. To the best of our knowledge, however, there has been no report describing changes of the choroidal structure in cases of PIOL after treatment. We also recently reported that EDI-OCT images can be converted to binary images, which can then be used to quantify the luminal and interstitial areas of the choroid [13–15]. We applied this technique to quantify the luminal and stromal areas of the choroid in eyes with PIOL. The purpose of this study was therefore to report changes of choroidal structure determined by binarization of the EDI-OCT images after treatment for PIOL.

## Methods

Five eyes of four patients (three men and one woman) with biopsy-diagnosed PIOL were examined by EDI-OCT before and 6 months after intravitreal methotrexate injections (IVMTXs). In addition, 15 eyes of 15 normal individuals controlled by age and refractive error were examined by EDI-OCT. Patients were evaluated and diagnosed in the Department of Ophthalmology of Tokushima University Hospital. Approval was obtained from the Institutional Review Board of Tokushima University Hospital to perform this study, and the patients gave their written informed consent prior to their inclusion in the study. The patients have provided permission to publish clinical data of their case in this study. Furthermore, the study adhered to the tenets of the Declaration of Helsinki. All patients underwent diagnostic pars plana vitrectomy, and vitreous samples were obtained during the vitrectomy. The diagnosis of PIOL was made by the detection of malignant lymphocytes, class IV or V, or an interleukin (IL)-10/IL-6 ratio > 1.0 in the vitreous fluid. Two eyes were treated with oral prednisolone before diagnostic vitrectomy, but EDI-OCT was not performed before steroid therapy.

The patient demographics and clinical findings are summarized in Table 1. The age of the patients ranged from 59 to 77 years (mean, 65.0 years). The PIOL in two patients was monocular, one patient had bilateral PIOL, and another patient had PIOL in the right eye and a history of PIOL in the left eye. One patient had a history of PCNSL. The cytological diagnoses were classes I, III, and V in one eye each, and were not available in two eyes. The IL-10/IL-6 ratio was > 1.0 in the vitreous of all eyes. Vitreous opacities were observed in all eyes. Retinal infiltrations and iritis were observed in two eyes each, and retinitis was present in one eye. The number of IVMTXs during 6 months ranged from 6 to 9 times (mean, 8.2 times). Before 6 months after the initial IVMTX, the level of IL-10 in the aqueous humor became undetectable in all eyes. The PIOL developed in a delayed fashion in the healthy fellow eye of one patient with unilateral PIOL, but this patient was dead of intracranial lymphoma before diagnostic vitrectomy and initiation of IVMTX. The diagnosis of this delayed-onset PIOL was made by the detection of an IL-10/IL-6 ratio > 1.0 in the aqueous fluid. The report of case 4 by Egawa et al. [12] has already been published.

**Table 1:** Clinical characteristics of eyes with PIOL

Case No.	Age Years/ Gender	Side	PCNSL prior to PIOL	Cytological diagnosis	Intraocular cytokine level			Ocular findings	Number of IVMTX	BCVA before IVMTX	BCVA after IVMTX
					IL-10 (pg/mL)	IL-6 (pg/mL)	Ratio of IL-10/IL-6				
1	59/M	OD	+	N/A	825	45	18.3	Iritis,OCV	9	1.2	1.5
		OS		N/A	5080	89.5	56.8	Iritis,OCV	9	0.6	0.9
2	67/F	OD	-	Class III	1870	1370	1.4	OCV, retinitis	8	0.9	0.9
3	77/M	OS	-	Class I	696	35.6	19.6	OCV, retinal infiltration (macula, periphery)	6	1.0	0.9
4	57/M	OD	-	Died before vitrectomy	225	22.4	10.0	OCV, retinal infiltration (posterior pole)	Died before IVMTX	0.4	N/A
		OS		Class V	3150	102	30.9	OCV, retinal infiltration (macula)	9	0.9	1.2

*BCVA* best-corrected visual acuity, *IL* interleukin, *IVMTX* intravitreal injection of methtorexate, *N/A* not available, *OCV* vitreous opacity, *OD* right eye, *OS* left eye, *PCNSL* primary central nervous system lymphoma, *PIOL* primary intraocular lymphoma

**Fig. 1:**
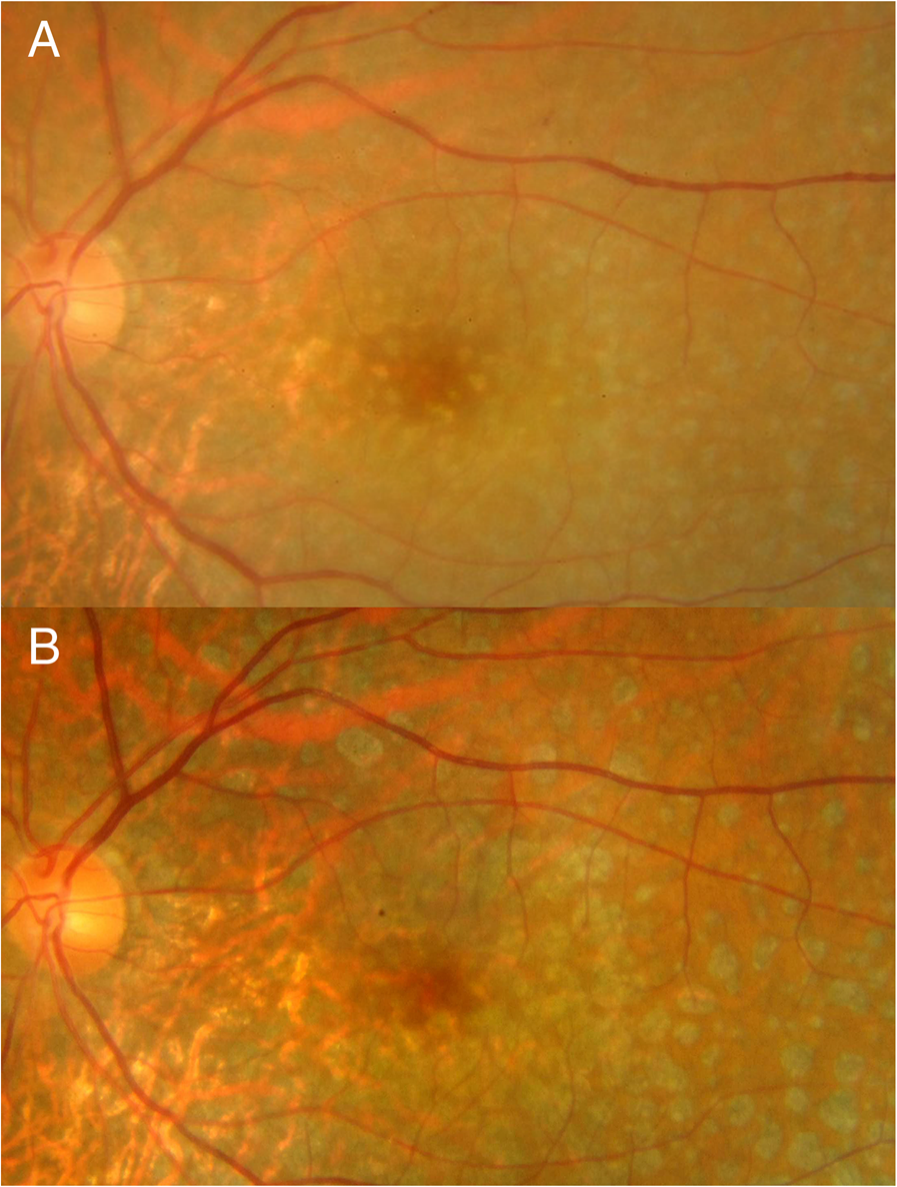
Fundus photograph in the left eye of case 4. **a** Before treatment, fundus photograph after diagnostic vitrectomy shows small yellowish lesions resembling drusen in the posterior fundus. **b** Six months after the initial intravitreal methotrexate injection, the yellowish lesions have changed to retinal pigment epithelial atrophy

All patients had a standard ophthalmologic examination before and after the IVMTX. The examination included measurement of best-corrected visual acuity, applanation tonometry, slit lamp biomicroscopy, indirect ophthalmoscopy, and color fundus photography (Fig. 1). Spectral-domain OCT (SD-OCT) was performed with the Heidelberg Spectralis (Heidelberg Engineering, Heidelberg, Germany). The subfoveal choroidal thickness was manually measured using the caliper function, and the subfoveal choroidal thicknesses on vertical and horizontal scans were averaged in each eye.

**Fig. 2:**
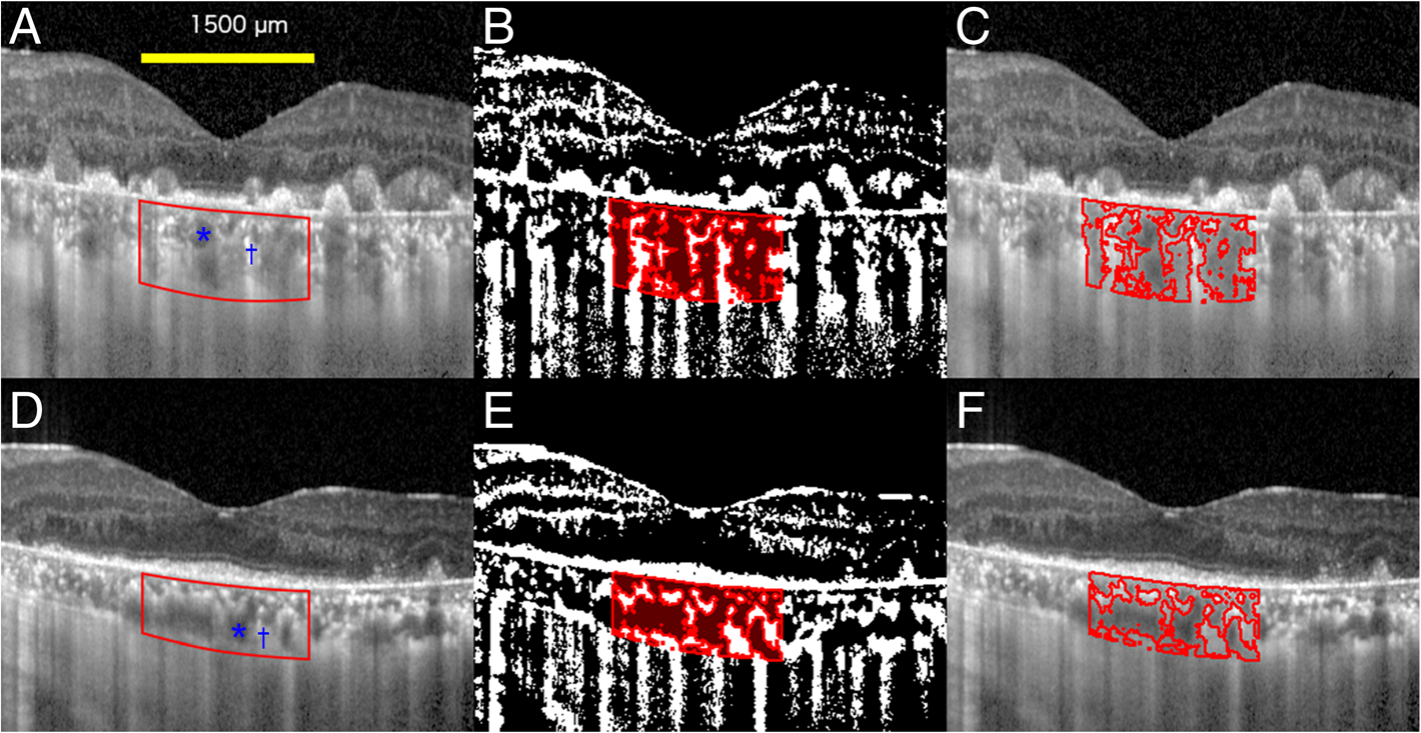
Enhanced depth imaging optical coherence tomographic (EDI-OCT) images and converted binary images in case 4. Upper row shows EDI-OCT images before treatment, and lower row shows images 6 months after the initial intravitreal methotrexate injection. EDI-OCT images through the fovea (**a**, **d**) were converted to binary images using ImageJ software. **a**, **d** The luminal area (dark area, asterisks) and the interstitial area (light area, daggers) can be seen. The examined area was determined to be 1,500 μm wide in the subfoveal choroid. It extended vertically from the retinal pigment epithelium to the chorioscleral border, and the choroidal area was set with the ROI manager of ImageJ. The rectangle surrounded by a red line was excised, and the dark areas were traced by the Niblack method. **b**, **e** Merged images of the binarized images and the margins of traced areas. In the binarized images, the light pixels were defined as the interstitial choroid or choroidal stroma, and the dark pixels were defined as the luminal area. **c**, **f** Merged images of the original EDI-OCT images and the margins of traced areas show that the traced areas coincide with the dark choroidal areas of the EDI-OCT image

Binarization of a choroidal area in the EDI-OCT image was done by a modified Niblack’s method as reported in detail (Fig. 2) [14]. Briefly, an EDI-OCT image was analyzed by ImageJ software (ImageJ, version 1.47, NIH, Bethesda, MD, USA). The examined area was 1,500 μm wide in the subfoveal choroid, and extended vertically from the retinal pigment epithelium (RPE) to the chorioscleral border. This choroidal area was selected with the ImageJ ROI Manager. Three choroidal vessels with lumens larger than 100 μm were randomly selected by the Oval Selection Tool on the ImageJ tool bar, and the average reflectivity of these areas was determined. The average brightness was set as the minimum value to minimize noise in the OCT image. Then, the image was converted to 8 bits and adjusted by the Niblack Auto Local Threshold. The binarized image was converted to the RGB image again, and the luminal area was determined using the Threshold Tool. The light pixels were defined as the interstitial areas, and the dark pixels were defined as the luminal areas. After adding the data of the distance of each pixel, the luminal and interstitial areas were automatically calculated.

Wilcoxon signed rank test was used to determine the significance of changes in the subfoveal choroidal thickness, interstitial area and luminal area. Mann–Whitney *U* test was used to compare the parameters in the 5 eyes with pretreatment PIOL and normal control eyes. All analyses were done with the SPSS version 22.0 (IBM Japan, Ltd., Japan) and the StatView software (Abacus; Abacus Concepts, Inc., Berkeley, California, USA). A *P* value of < 0.05 was considered statistically significant.

## Results

Subfoveal choroidal thickness and areas in EDI-OCT images before and after IVMTX are shown in Table 2. The subfoveal choroidal thickness 6 months after the initial IVMTX (182.7 ± 39.3 μm) was significantly decreased from that before IVMTX (215.6 ± 38.1 μm, *P* = 0.0431). In the binarized images, the interstitial area of the 1500-μm-wide subfoveal choroid after treatment (0.1088 ± 0.0167 mm^2^) was significantly decreased compared with that before treatment (0.1240 ± 0.0200 mm^2^, *P* = 0.0431), while the luminal area was not significantly changed after IVMTX (0.1942 ± 0.0403 mm^2^ before treatment, 0.2007 ± 0.0439 mm^2^ after treatment; *P* = 0.8927). In addition, the pretreatment interstitial area was higher in the affected eyes compared with the healthy fellow eyes in cases 3 and 4 with unilateral PIOL. However, the interstitial area was not higher in the affected eye compared with the fellow eye with a history of PIOL in case 2. Notably, the interstitial area and choroidal thickness were increased after development of PIOL in the right eye of case 4, while the luminal area was not changed.

**Table 2:** Subfoveal choroidal area and thickness in EDI-OCT images before and after IVMTX

Case No.	Side	Status	Time of examination	Interstitial area (mm^2^)	Luminal area (mm^2^)	Choroidal thickness (μm)
1	OD	PIOL(+)	Pre-IVMTX	0.1371734	0.2060821	224.0
			Post-IVMTX	0.1264265	0.2448991	211.5
	OS	PIOL(+)	Pre-IVMTX	0.1364545	0.2145141	220.5
			Post-IVMTX	0.1207003	0.2155650	204.0
2	OD	PIOL(+)	Pre-IVMTX	0.0903745	0.1277632	150.0
			Post-IVMTX	0.0849373	0.1390663	118.0
	OS	History of PIOL		0.9527313	0.1195278	158.0
3	OD	Normal		0.1061557	0.3154791	264.0
	OS	PIOL(+)	Pre-IVMTX	0.1353078	0.2085307	238.0
			Post-IVMTX	0.1120500	0.1980837	207.5
4	OD	Normal → PIOL(+)	Pre-PIOL	0.0741932	0.2257468	184.0
			Post-PIOL	0.1089445	0.2276595	212.5
	OS	PIOL(+)	Pre-IVMTX	0.1204610	0.2466626	245.5
			Post-IVMTX	0.0997601	0.1735233	172.5

*EDI-OCT* enhanced depth imaging optical coherence tomography, *IVMTX* intravitreal injection of methtorexate, *OD* right eye, *OS* left eye, *PIOL* primary intraocular lymphoma, *Pre-PIOL* before development of primary intraocular lymphoma, *Post-PIOL* after development of primary intraocular lymphoma

There was no significant difference in the mean age and refractive error between the patients and controls (*P* = 0.8958, *P* = 0.9652, respectively). The interstitial area and choroidal thickness were significantly increased in the 5 eyes with pretreatment PIOL (0.1240 ± 0.0200 mm^2^, 215.6 ± 38.1 μm, respectively) compared with the normal control eyes (0.1006 ± 0.0157 mm^2^, *P* = 0.0207; 174.4 ± 45.1 μm, *P* = 0.0495; respectively), while the luminal area was not significantly different (*P* = 0.2752).

## Discussion

The present study showed that eyes with PIOL had a markedly increased interstitial area of the choroid compared with healthy fellow eyes. In addition, the interstitial area in the affected eye was not increased compared with the fellow eye with a history of PIOL. Consistent with our findings, the results of gross examination of the enucleated eye with PIOL showed a thickened choroid as well as multiple white areas of retinal and subretinal tumor infiltration [16]. The present study also showed that choroidal thickness and the interstitial area were significantly decreased after treatment for PIOL. After delayed onset of PIOL, increased interstitial area, thickened choroid and unchanged luminal area were observed in one eye. The interstitial area and choroidal thickness were significantly increased in the eyes with pretreatment PIOL compared with the normal control eyes. Choroidal thickness and interstitial area on EDI-OCT images may have corresponded with an increase in disease activity. Thus, there is a possibility that EDI-OCT is useful for monitoring the progression or regression of PIOLs, which may then influence the management of PIOL cases.

The mechanism of the increased interstitial area of the choroid in PIOL remains unknown. However, histopathological studies indicated that accumulation of reactive inflammatory cells in the choroid may contribute to the increased choroidal interstitial area [1, 17, 18]. Reactive inflammatory cells are commonly mixed with malignant lymphoma cells in eyes with PIOL [17]. Lopez et al. [18] reported an immunohistochemical examination of enucleated globes with PIOL. They found malignant B cells in the retina, whereas reactive T-cells were concentrated in the choroid and maintained a CD4/CD8 ratio of 2:1, which is typical of reactive T cell processes. Read et al. [17] reported results of a chorioretinal biopsy from a 54-year-old woman with PIOL who lost vision in her left eye. Pleomorphic, malignant lymphocytes and necrotic debris were detected in the subretinal space, and the choroid was infiltrated with reactive inflammatory cells. Chan et al. [1] reported results of immunohistochemistry for enucleated eyes with PIOL, showing an intense expression of chemokine receptors, CXCR4 and CXCR5, with malignant lymphoma in the retinal and subretinal areas, but without lymphocytes infiltrating the choroid. The presence of T-cells in the choroid was postulated to reflect an immune response against the tumor, preventing it from penetrating Bruch’s membrane and extending into the choroid [17, 18].

Another possible mechanism explaining the increased interstitial area is direct invasion of lymphoma cells into the choroid. Chan et al. [19] reported a murine model of PIOL, where lymphoma cells were inoculated into the vitreous of adult immunocompetent mice, then the eyes were harvested. In the early period after inoculation, tumor cells from the vitreous migrated through the retina and accumulated between the RPE and sensory retina. More than 2 months after inoculation, lymphoma cells rarely broke through the RPE and infiltrated the choroid and sclera. Jang et al. [4] reported longitudinal SD-OCT changes in eyes with PIOL. In their study, hyperreflective signals appearing as nodules or bands were found across the retina, RPE, and/or choroid. In one of five patients with PIOL, hyperreflective foci were found in the choroid as well as retina. Thus, direct invasion of lymphoma cells into the choroid may rarely contribute to the increased choroidal interstitial area.

The decrease in choroidal thickness and interstitial area after treatment for PIOL may be due to a decrease in reactive inflammatory cells. However, cell toxicity of intravitreally injected methotrexate may also contribute to the decrease in choroidal thickness and interstitial area.

SD-OCT and fundus autofluorescence (FAF) findings in eyes with PIOL were recently reported, and these imaging techniques were shown to be useful for diagnosing and determining the activity of PIOL. Casady et al. [7] reported that the nodular hyperreflective spots in the OCT images were present at the level of the RPE in 6 of 14 eyes (43 %) with PIOL. Jang et al. [4] reported that the hyperreflective signals were seen as dots, nodules, or bands throughout the retina and RPE in eyes with PIOL. In their study, the number of hyperreflective foci in the inner retina increased with increased activity of PIOL.

Casady et al. [7] showed that the hyperautofluorescence spots on FAF images changed to hypoautofluorescent spots corresponding to the atrophic areas in the RPE after treatment for PIOL. Similarly, we have reported that the FAF images had a granular pattern of slight hypoautofluorescence and hyperautofluorescence before treatment and patchy hypoautofluorescence corresponding to RPE atrophy after the treatments [12]. These differences in the FAF findings may be useful for determining the sites of PIOL activity.

This study has limitations. We studied only five eyes with PIOL, and further studies examining a larger number of cases treated for PIOL will be needed to accurately determine choroidal changes after treatment. In addition, further studies examining whether choroidal interstitial area increased with increased PIOL activity will be necessary.

## Conclusions

EDI-OCT and binarization of the EDI-OCT images may be a useful and noninvasive method to evaluate the choroid before and after treatment for PIOL. After treatment for PIOL, the EDI-OCT images showed a thinner choroid and binarization of the EDI-OCT images showed markedly decreased interstitial areas compared with the luminal areas. After delayed onset of PIOL, increased interstitial area, thickened choroid and unchanged luminal area were observed in one eye. In addition, the interstitial area was higher before treatment in the affected eye compared with the healthy fellow eye. The interstitial area and choroidal thickness were significantly increased in the eyes with pretreatment PIOL compared with the normal control eyes, while the luminal area was not significantly different. The binarized EDI-OCT images can provide useful information on choroidal structure in eyes with PIOL, and combining these images with intraocular IL levels or FAF images should provide valuable information for determining the PIOL activity, which may influence treatment decisions for managing the disease.

## Abbreviations

CNS: Central nervous system
EDI-OCT: Enhanced depth imaging optical coherence tomography
FAF: Fundus autofluorescence
IL: Interleukin
IVMTX: Intravitreal methotrexate injection
non-PCNSL: non-primary central nervous system lymphoma
OCT: Optical coherence tomography
PCNSL: Primary central nervous system lymphoma
PIOL: Primary intraocular lymphoma
RPE: Retinal pigment epithelium
SD-OCT: Spectral-domain optical coherence tomography
